# Non-linear dependency between spiking response and gamma-band power of local field potentials in the human auditory cortex

**DOI:** 10.1186/1471-2202-14-S1-P175

**Published:** 2013-07-08

**Authors:** Hiroyuki Oya, Kirill V Nourski, Ariane E Rhone, Hiroto Kawasaki, Matthew A Howard

**Affiliations:** 1 Department of Neurosurgery, University of Iowa, Iowa City, IA, 52242, USA

## 

Recent advancement in recordings of extracellular potentials from multiple sites in the human brain [1] has allowed us to simultaneously record spiking activity and local field potentials (LFP) to examine the brain's response to experimental stimuli. Various mechanisms have been suggested for the generation of the LFP. A point of special interest is that LFP reflects not only the inputs to the tissue, but also local processing. For example, spike contribution to the high-frequency LFP through increased spike rate or increased synchrony has been suggested [2]. Subjects (n = 19 ) were epilepsy patients who needed intracranial electrode implantation and chronic monitoring of for diagnostic and treatment purposes. Protocols were approved by University of Iowa IRB. Recordings were made from micro contacts implanted in the Heschl's gyri during pure tone presentations. Single-trial gamma-band responses were extracted using band-pass filters (40-150 Hz). Spike trains at the same contacts were analyzed with Poisson generalized linear models (GLM) with coded stimulus convolved with finite impulse-response (FIR) function which was estimated from the data, as well as spiking history (up to 80 ms duration, determined by minimum BIC criterion) as covariates. We treated the system as a time-invariant black box and the relationship between spiking activity (rates) and gamma-band LFP power was analyzed with Volterra kernel expansion (memory length of 200 ms) up to the second-order to examine potentially non-linear dependencies [3] (Figure 1A). Estimated discharge rates were used as the inputs and gamma LFP power was used as the outputs of the system. Weighted least-squares support-vector machines were utilized for estimating the regression coefficients [4], and the kernels were expanded using Laguerre orthogonal basis. First order kernel (linear filter) typically decreased toward zero within 150 ms lag time (Figure 1A and 1B), and 2^nd ^-order kernels typically contain negative components, indicating non-linear suppressive effect on the gamma. (Figure 1B). A separate correlation analysis between spike count and gamma-band power within post-stimulus 200 ms shows strong positive correlation (Figure 1C).

**Figure 1:**
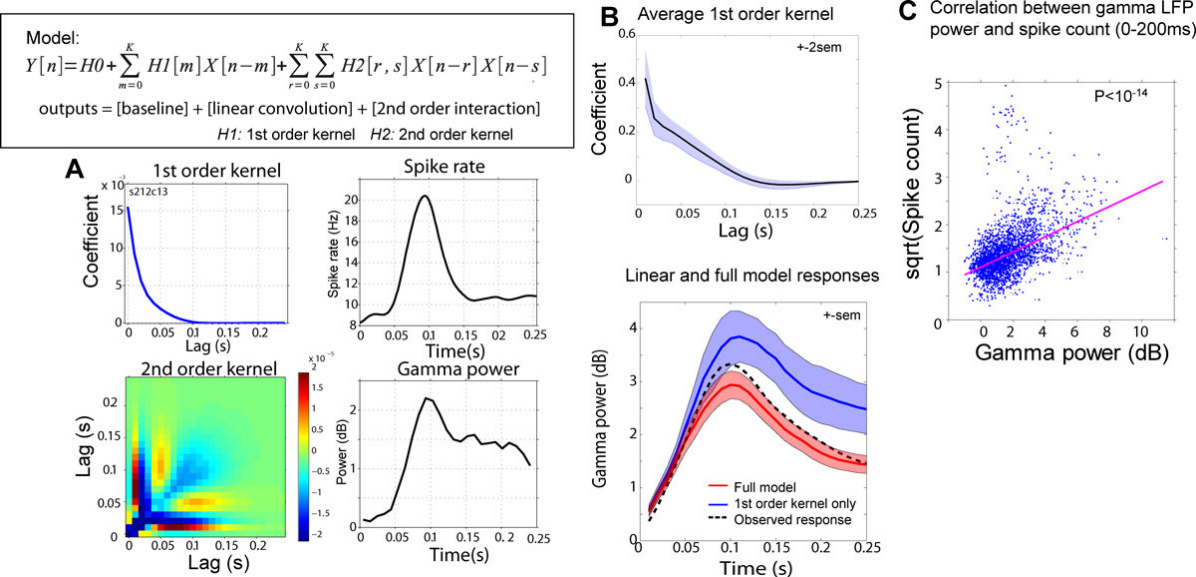


## Conclusions

Gamma-band LFP power correlates with spiking activity in human auditory cortex. Although, the detailed time course of gamma-band LFP cannot be explained by linear filtering of the spiking activity. A higher-order no-linear relationship between the two is suggested.
